# Prospects for Electrical Performance Tuning in Ca_3_Co_4_O_9_ Materials by Metallic Fe and Ni Particles Additions

**DOI:** 10.3390/ma14040980

**Published:** 2021-02-19

**Authors:** Gabriel Constantinescu, Sergey M. Mikhalev, Aleksey D. Lisenkov, Daniela V. Lopes, Artur R. Sarabando, Marta C. Ferro, Tiago F. da Silva, Sergii A. Sergiienko, Andrei V. Kovalevsky

**Affiliations:** 1 Department of Materials and Ceramics Engineering, CICECO–Aveiro Institute of Materials, University of Aveiro, 3810-193 Aveiro, Portugal; lisenkov@ua.pt (A.D.L.); daniela.rosendo.lopes@ua.pt (D.V.L.); artursarabando@ua.pt (A.R.S.); marta.ferro@ua.pt (M.C.F.); tiagofvs@ua.pt (T.F.d.S.); sergiienko@ua.pt (S.A.S.); akavaleuski@ua.pt (A.V.K.); 2 TEMA-NRD, Mechanical Engineering Department, Aveiro Institute of Nanotechnology (AIN), University of Aveiro, 3810-193 Aveiro, Portugal; mikhalev@ua.pt

**Keywords:** thermoelectric cobaltites, electrical performance, composite approach, transition metals additions, controlled interactions

## Abstract

This work further explores the possibilities for designing the high-temperature electrical performance of the thermoelectric Ca_3_Co_4_O_9_ phase, by a composite approach involving separate metallic iron and nickel particles additions, and by employing two different sintering schemes, capable to promote the controlled interactions between the components, encouraged by our recent promising results obtained for similar cobalt additions. Iron and nickel were chosen because of their similarities with cobalt. The maximum power factor value of around 200 μWm^−1^K^−2^ at 925 K was achieved for the composite with the nominal nickel content of 3% vol., processed via the two-step sintering cycle, which provides the highest densification from this work. The effectiveness of the proposed approach was shown to be strongly dependent on the processing conditions and added amounts of metallic particles. Although the conventional one-step approach results in Fe- and Ni-containing composites with the major content of the thermoelectric Ca_3_Co_4_O_9_ phase, their electrical performance was found to be significantly lower than for the Co-containing analogue, due to the presence of less-conducting phases and excessive porosity. In contrast, the relatively high performance of the composite with a nominal nickel content of 3% vol. processed via a two-step approach is related to the specific microstructural features from this sample, including minimal porosity and the presence of the Ca_2_Co_2_O_5_ phase, which partially compensate the complete decomposition of the Ca_3_Co_4_O_9_ matrix. The obtained results demonstrate different pathways to tailor the phase composition of Ca_3_Co_4_O_9_-based materials, with a corresponding impact on the thermoelectric performance, and highlight the necessity of more controllable approaches for the phase composition tuning, including lower amounts and different morphologies of the dispersed metallic phases.

## 1. Introduction

Nowadays, there is a great need for competitive renewable energy sources and devices, and many efforts are being put into their large-scale research, development, and innovation. Among the various existing types and in the context of the present environmental challenges, thermoelectric (TE) generation stands out as one of the most promising options allowing for direct conversion of waste heat into electrical power [1,2,3,4], with no unwanted byproducts or side effects. The TE materials making up the TE generators can achieve this impressive feat directly, in a solid state, thanks to the Seebeck effect [3]. TE technology is autonomous, reliable, scalable, robust, has no moving parts, and requires virtually no maintenance, making it ideal for mobile and/or remote applications [5,6]. The relatively low conversion efficiencies of the most established TE materials significantly limit their possible number and range of applications [7], although promising new prospects are outlined with the recent aid of machine learning and artificial intelligence tools [8]. The performance of a TE material is always limited by the Carnot efficiency [9] and is measured by the dimensionless figure-of-merit ZT = (α^2^ σ)/κ T, where α is the absolute Seebeck coefficient, σ is the electrical conductivity, κ is the total thermal conductivity, and T is the prospective working temperature. The electrical part of ZT, (α^2^ σ), represents the power factor (PF) and depends entirely on the material’s intrinsic physical properties.

Established TE materials have ZT ≈ 1 and include PbTe, Bi_2_Te_3_, Bi_2_Se_3_ [10], skutterudite [11], half-Heusler alloys [12], intermetallic Zintl phases [13], and some Si-based alloys [14]. Most of them are narrow band-gap semiconductors and have optimum performances at low and intermediate working temperature ranges. Because of some limitations/drawbacks (degradation and decomposition at temperatures above ~500–600 °C, thermal and/or chemical instability, expensive, toxic, and/or scarce constituents) [15], they are not suitable for waste heat recovery applications and their use is restricted to some few, niche applications [3].

Relatively recently, large band-gap oxide semiconductors have attracted attention for potential power generation applications [16]. The discovery for the first time in 1997 of attractive TE properties in Na_x_CoO_2_ ceramics [17], led to other compositions based on transition metal oxides with promising TE properties and important intrinsic advantages (abundance, low environmental impact, moderate reactivity and high thermo-chemical stability in broad high-temperature ranges) over the ‘classical’ TE materials [18,19], to be considered for power generation applications at high temperatures and in oxygen-rich environments [20,21]. Furthermore, many of these oxides (e.g., titanium oxides [22], manganese oxides [23], nickel oxides [24], iron oxides [25], zinc oxides [26]) have been found to have adaptative crystal structures [27].

The Ca_3_Co_4_O_9_ compound is considered to be one of the most promising p-type TE oxide to date, suitable for waste heat recovery applications in air, at high temperatures [28]. The main challenges for this material are related to its strong anisotropic electrical properties induced by the ‘misfit layered’ crystal structures and its relatively low bulk density and weak mechanical strength (caused by the big difference between the maximum stability temperature of the Ca_3_Co_4_O_9_ phase and the corresponding solidus temperature). These issues severely limit its use in power generation applications and technologies.

The Ca_3_Co_4_O_9_ oxide is intrinsically nanostructured and has a monoclinic crystal structure (P2(3) space group) consisting of two different monoclinic alternating layers, stacked in the *c*-axis direction. A distorted triple rock-salt (RS) type [Ca_2_CoO_3_] insulating layer (having Co^2+^ cations) is sandwiched between two hexagonal (H) CdI_2_-type [CoO_2_] conductive layers (consisting of edge-shared [CoO_6_] octahedra) where the mean cobalt valence is between (3+) and (4+), building a complex misfit structure along the *b*-axis direction [29,30], since the *b* cell parameters are incommensurate. In contrast, the lattice parameters in the *a* and *c* directions for both layers are equal. Based on its polycrystalline structure, the Ca_3_Co_4_O_9_ compound is more technically written sometimes with the chemical formula [Ca_2_CoO_3_][CoO_2_]_1.62_, where ‘1.62’ is the incommensurability ratio (*b*_RS_/*b*_H_), found to be responsible for the high Seebeck coefficient values [31], among other things.

The TE performance of Ca_3_Co_4_O_9_ materials is usually enhanced using cation (partial) substitutions in one or both crystal sublattices [32,33], micro- and nanostructural engineering techniques [34,35] and some composite approaches [36,37]. The material’s density and grain connectivity is often controlled and increased through texturing and densification methods like spark plasma sintering, SPS [38] and laser floating zone melting, LFZ [39], and through the use of high-quality and high-reactivity, ultrafine precursor powders obtained by ‘wet chemistry’ methods [40,41].

Two-step sintering schemes also result in quite dense ceramics with improved electrical performance, but require long processing/annealing times to stabilize the TE phase [39,42,43] and can give rise to undesired secondary phases. According to the equilibrium phase diagram of the Ca–Co–O system in air [44,45], the Ca_3_Co_4_O_9_ phase is stable up to 1199 K (926 °C), After this point, it decomposes to Ca_3_Co_2_O_6_ and CoO, which are both stable up to 1299 K (1026 °C). Following the stoichiometry line further shows the first liquid phase appearing at 1623 K (1350 °C), the fact which is very important to consider for issues related to the bulk density and/or porosity of the Ca_3_Co_4_O_9_ phase.

It has been reported that partial substitutions with Fe and Ni for Co in Ca_3_Co_4_O_9_ may increase the electrical conductivity by increasing the holes concentration from the conductive layers, provided that Fe and Ni take the usual oxidation states (2+) and/or (3+) [33,46], smaller than that of the mean Co valence responsible for the good electrical conductivity from this material, between (3+) and (4+) [29]. This fact was observed for small substitutions. For higher substitutions, however, the opposite effect was observed [47]. The ionic radii of the substitutional cations also play an important role in the electrical conduction from Ca_3_Co_4_O_9_, due to crystal distortion and scattering effects. Furthermore, it has been recently reported that at low concentrations the iron prefers to substitute cobalt, while at high Fe additions it may even substitute calcium [48]. Fe K-edge XANES spectra have shown that Fe may select different doping sites in the Ca_3_Co_4_O_9_ system. Fe can substitute Ca in both Ca1 and Ca2 sites, and can also substitute Co in the Co1 sites from the RS-type layer [48]. Nevertheless, it has been shown that the preferred doping sites are Ca1 and Co1 from the RS-type layer [48].

The present research work takes advantages of the previously published results for metallic Co additions [49] and reports on a similar approach for improving the high-temperature electrical performance of bulk Ca_3_Co_4_O_9_ materials, by similar metallic Fe and Ni additions and employment of two different sintering schemes, for producing porous and dense Ca_3_Co_4_O_9_-based composites. The degree of success of this approach is measured by the electrical counterpart of the TE performance (electrical conductivity, Seebeck coefficient, and resulting power factor) of the resulting composites, which are compared to the best ones obtained for the Co additions and to some values reported in literature, being related to the prepared samples’ phase compositions, morphologies, and microstructural features. As an initial goal, we aimed for a beneficial porosity filling effect provided by the oxidation of the added metallic particles under the two sintering schemes employed. Additional beneficial effects were expected from possible substitutions in the conductive layers and/or formation of useful secondary phases.

## 2. Materials and Methods

Single-phase Ca_3_Co_4_O_9_ ceramic materials used as matrices for the (1 − x) Ca_3_Co_4_O_9_/xFe or Ni (x = 0%, 3%, 6% and 9% vol.) compositions were prepared through a ‘wet-chemistry’ modified Pechini method [49]. Appropriate amounts of micrometric Fe and Ni particles and Ca_3_Co_4_O_9_ powders were mixed in an agate mortar, in ethanol, to produce the desired compositions. Pristine matrix samples were kept as a reference.

The powders have been uniaxially pressed at 200 MPa (15.7 kN). The pelletized samples were subsequently sintered in air, using two distinct sintering approaches, similar to Ref. [49]. The one-stage (1ST) route includes one heating step to 1173 K (2 K/min), followed by a 24 h dwell time and then a slow cooling (2 K/min) to room temperature (RT). The two-stage (2ST) scheme providing higher densification includes a first heating step to 773 K (8 K/min), followed by a second heating step to 1473 K (2 K/min), then a 6 h dwell time at this temperature, followed by a fast cooling down to 1173 K (10 K/min), where this temperature is kept for 72 h, and finally, cooling down to room temperature, slowly (2 K/min).

After sintering, the resulted pellets were polished and finely ground or cut in the adequate shapes and sizes, for the relevant characterizations to be performed onward. The experimental densities (*ρ_exp_*) of Ca_3_Co_4_O_9_-based ceramics were determined by geometrical measurements and weighing (masses over volumes). The estimated errors in all cases were found to be <3% (~0.03 g/cm^3^). Whenever possible, the *ρ_exp_* values were compared to the theoretical density (*ρ_th_*) values of the respective composites, calculated from the sum of the products of the theoretical densities of the reference intensity ratio-estimated phases (from X-Ray Diffraction analyses) and their respective amounts, in each case, i.e., by a simple mixing rule, using the following theoretical density values: *ρ_th_*(Ca_3_Co_4_O_9_) = 4.69 g/cm^3^, from [50] and PDF card #04-016-0860; *ρ_th_*(Ca_3_Co_2_O_6_) = 4.5 g/cm^3^, from PDF cards #00-051-0311 [51] and #04-010-0812; *ρ_th_*(CoO) = 6.58 g/cm^3^, from PDF card #04-005-4395.

The phase identification was performed through X-Ray Diffraction (XRD) analyses, for the various powdered Ca_3_Co_4_O_9_-based samples, after both sintering cycles, at RT, using a PHILIPS X’PERT system with CuK_α_ radiation (Cu_α_ = 1.54060 Å), with 2θ angles ranging between 5 and 90° and a step and exposure time of 0.02° 2θ and 3 s, respectively. The phase content was estimated using the reference intensity ratio (RIR) method [52], using the Panalytical HighScore Plus 4.1 (PDF-4) software. The strongest peaks from each phase were used for analysis, and the corresponding scale factors were taken from the PDF-4 database. Morphological and microstructural characterizations of representative selected fractured samples coated with carbon were performed with a scanning electron microscope (SEM, Hitachi SU-70 instrument (Aveiro, Portugal)), equipped with an energy-dispersive X-Ray spectroscopy (EDX, Bruker Quantax 400 detector (Aveiro, Portugal) module.

Simultaneous electrical conductivity (σ) and Seebeck coefficient (α) measurements have been performed on selected rectangular bar-shaped samples (~10 mm × 2 mm × 2 mm), in constant air flow, from 475 K to 975 K, with a step of 50 K, employing a steady-state technique and using a custom experimental setup described in detail elsewhere [53]. Freshly cut samples of each composition have been fixed inside a specially designed alumina sample holder, placed inside a high-temperature furnace, one horizontally (*σ* sample, electrically connected with fine Pt wires, following a four-point probe direct current, DC technique arrangement) and the other vertically (α sample, subjected to a local constant temperature difference of ~14 K). The estimated experimental error in measured values did not exceed 3–5% for the conductivity and 5–7% for Seebeck coefficient. The PF values have been calculated from the measured *σ* and *α*, in each case, at each temperature step.

The various plots have been constructed using the OriginPro 8.5 software (Northampton, MA, USA).

## 3. Results and Discussions

### 3.1. The Samples Processed under the 1ST Route

#### 3.1.1. Compositional and Morpho-Structural Evolution

The following Table 1 lists the denominations of the samples and their relevant properties, related to the phase composition, density, and electrical properties.

**Figure 1:**
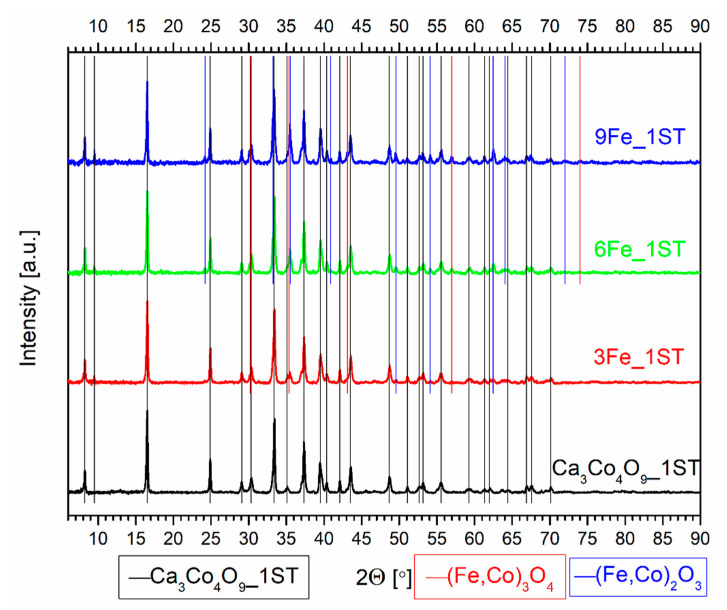
Normalized XRD patterns of the 1ST sintered reference matrix and Fe added samples. The vertical lines of different colors mark the main identified phases.

**Figure 2:**
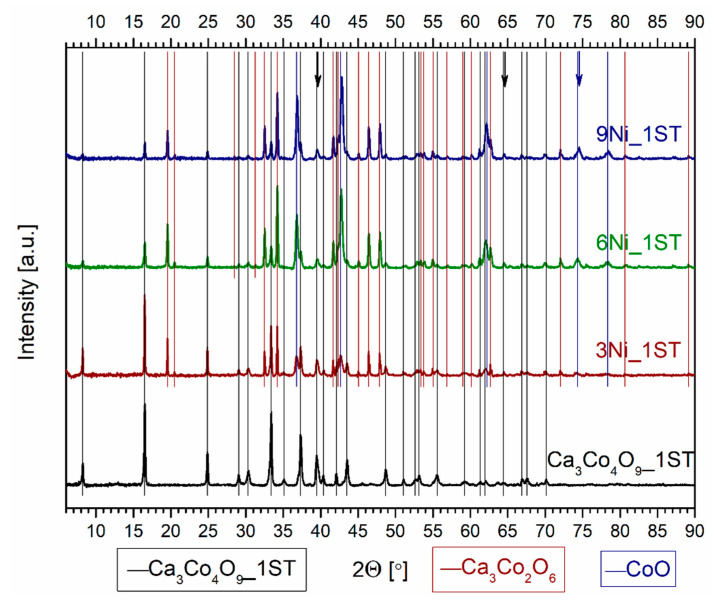
Normalized XRD patterns of the 1ST sintered reference matrix and Ni added samples. The vertical lines of different colors mark the main identified phases. The arrows mark the peak displacements of the phases suggesting substitution with Ni, namely Ca_3_Co_4_O_9_ (black arrows) and CoO (blue arrow).

The evolution of the phase composition for all 1ST sintered samples can be clearly seen in the corresponding XRD patterns from Figure 1 and Figure 2, where the various vertical lines of different colors mark the identified phases, and the arrows from Figure 2 mark the only peak displacements found, for the Ni added samples. The diffraction pattern for the single-phase reference matrix, Ca_3_Co_4_O_9__1ST, is shown for comparison in both figures, marked by the vertical black lines, in agreement with the work of Masset et al. [29] and with other literature references [44,45]. The wide peaks representing the various crystal planes are typical for this material sintered in the described conditions [40]. The XRD patterns for the samples with Fe and Ni additions clearly show the presence of additional phases, indicated by the vertical lines of different colors, following the legends from the bottom of the figures.

The secondary phases from the iron-added samples include the solid solutions (Fe,Co)_3_O_4_ and (Fe,Co)_2_O_3_, with different transition metal oxidation states and ratios, their concentration increasing in proportion with the amount of Fe (as depicted in Figure 1 and Table 1). The formation of additional secondary phases in Fe-substituted Ca_3_Co_4_O_9_ samples is also reported elsewhere in the literature, for various Fe contents ([54] and references therein). In any case, the major phase in all Fe-containing samples was found to be Ca_3_Co_4_O_9_, probably containing some minor amount of incorporated iron. Although it is generally accepted that iron substitutes cobalt in CoO_2_ and/or Ca_2_CoO_3_ layers, there are indications that iron may also substitute calcium cations [54]. The phase decomposition, promoted by the iron additions, leads to the appearance of excessive porosity in the 1ST sintered samples (Table 1), in contrast to the pore filling effect provided by the cobalt additions [49].

The evolution of the phase composition in the Ni-added samples shows notably different trends (Figure 2). The addition of metallic Ni particles does not promote the formation of any major Ni-containing secondary phases and apparently results in the substitution in the TE Ca_3_Co_4_O_9_ phase (less) and the secondary CoO phase (more). This fact is indicated by the arrows from Figure 2, marking the shifts to higher 2θ angles of some peaks belonging to Ca_3_Co_4_O_9_ (black arrows) and CoO (blue arrow). One possible explanation for this preferential formation of the CoO phase is the total mutual solubility between CoO and NiO at 1173 K [55]; this phase is expected to be more stable than Ni-substituted Ca_3_Co_4_O_9_ and/or Ca_3_Co_2_O_6_. Ca_3_Co_2_O_6_ was also detected in the Ni-containing samples, being the major phase in the 6 and 9Ni_1ST samples. While the relative amounts of Ca_3_Co_4_O_9_ and Ca_3_Co_2_O_6_ phases appear to stabilize in the 6 and 9Ni_1ST samples, the CoO phase content increases from 3Ni_1ST to 9Ni_1ST (Table 1). All Ni-added samples possess higher relative densities than that of the pure matrix reference. It is worth pointing out that among all samples, the 3Ni_1ST samples have the highest density value (2.85 g/cm^3^) for the largest amount of Ca_3_Co_4_O_9_ phase (67 wt.%). Still, the low preservation of the main Ca_3_Co_4_O_9_ thermoelectric phase represents a significant disadvantage of the studied approach in this case, as compared to the pore filling effects promoted by the cobalt additions [49].

**Figure 3:**
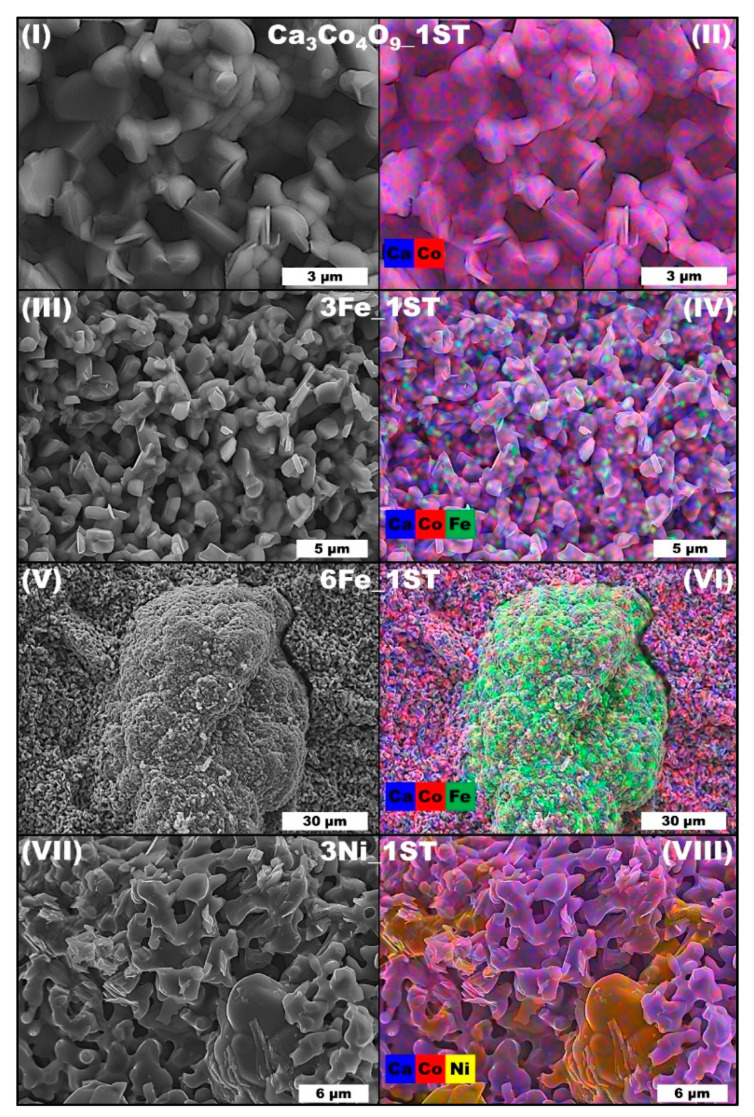
Representative SEM micrographs (**I**,**III**,**V**,**VII**) and EDS maps (**II**,**IV**,**VI**,**VIII**) of selected 1ST sintered samples (fractures).

The microstructural characterization results for the 1ST samples shown in Figure 3 mainly support the previous XRD findings and obtained density values. Unlike the more evident pore-filling effect observed previously for Co additions [49], the Fe and Ni ions from this study can be found predominantly in the two different iron and cobalt containing solid solutions (Figure 3IV,VI) and in the Ca_3_Co_4_O_9_ and CoO phases (Figure 3VIII), respectively, providing only a marginal porosity-filling effect in the corresponding composites, upon oxidation in air at 1173 K. Only the 3Ni_1ST samples apparently show a somewhat similar pore-filling effect (Figure 3VIII), provided by the Ni-doped CoO grains. For these latter samples, the Ca_3_Co_4_O_9_ and CoO phases are easily distinguishable by their different characteristic morphologies (Figure 3VIII). The morphology of the Ca_3_Co_4_O_9_ grains (pure and substituted) remains essentially unchanged in all samples. The small grain sizes and low particle size dispersion, characteristic for the Ca_3_Co_4_O_9_ phase obtained by the combustion-based modified Pechini method, as well as the preferential crystal growth along the *a-b* crystallographic plane, can also be seen in Figure 3, where the mean Ca_3_Co_4_O_9_ grain sizes are estimated to be around 1 μm in the planar direction (*a-b* plane), and ~0.5 μm in thickness (along the *c*-axis). This apparent thickness value is given by the stacking of the very-thin plate-like grains of Ca_3_Co_4_O_9_, each one having a thickness of ~35 nm, as it was estimated for similar cobalt oxides using Scherrer’s formula [56]. On the other hand, the Fe and Ni-rich phases can be distinguished by the rather larger agglomerates (Figure 3VI,VIII).

The trends observed in the compositional and microstructural evolution from all samples are further correlated with their electrical properties.

#### 3.1.2. Electrical Performance

**Figure 4:**
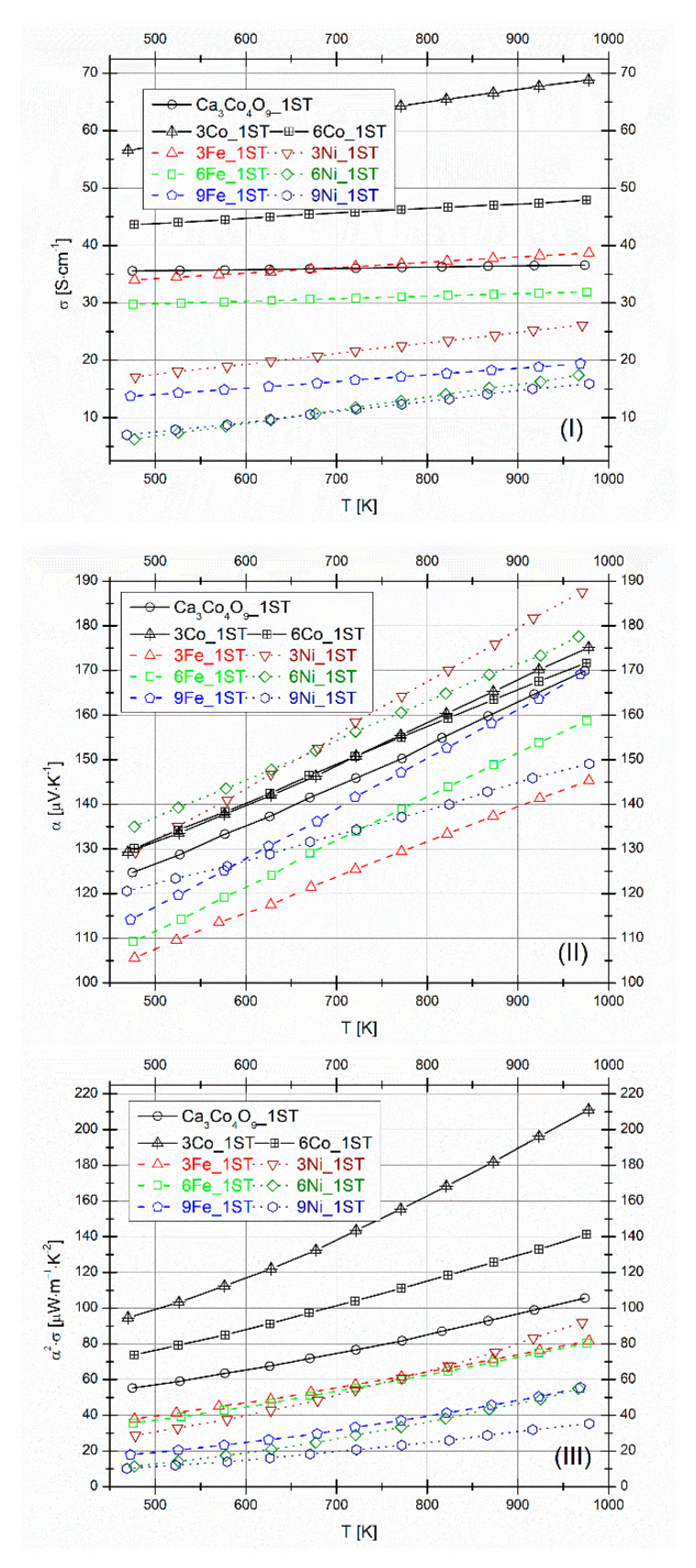
Electrical conductivity (**I**), Seebeck coefficient (**II**), and power factor (**III**) for 1ST sintered samples. The results for selected Co additions are presented for comparison.

The evolution of the electrical conductivity (*σ*), Seebeck coefficient (*α*), and power factor (*α^2^·σ*) with temperature for all 1ST-sintered samples is shown in Figure 4I–III, respectively. The previously obtained results for selected Co additions (3 and 6 vol.%) [49] are plotted for comparison.

All σ values increase linearly with temperature, in the whole measured temperature range, following different slopes (Figure 4I). This trend corresponds to a semiconducting-like behavior (*dσ/dT* ≥ 0) and is typical for this material at high temperatures (conduction is done along the *c*-axis direction [29]), also found elsewhere in literature, for similar cases [57,58,59]. With respect to the pure matrix reference samples, the highest σ values are still measured for the 3 and 6Co_1ST samples, reported in our previous work [49]. Between the different Fe and Ni added samples, however, only the 3 and 6Fe_1ST samples have high σ values (≥30 Scm^−1^), close to the ones measured for the pure matrix reference sample (~35 Scm^−1^). Nevertheless, these values measured for the 3 and 6Fe_1ST samples are higher than some of the best-reported values from the literature, for high-density SPS consolidated Fe-doped Ca_3_Co_4_O_9_ samples (~20 Scm^−1^) [54]. Furthermore, for the case of Ni additions, the best *σ* values measured for the 3Ni_1ST samples (17–27 Scm^−1^) are very close to some of the best reported values from literature (20–24 Scm^−1^), for Ni-doped Ca_3_Co_4_O_9_ samples prepared and measured in similar conditions [46]. In any case, it has been reported that lower substitutions of Fe and Ni in Ca_3_Co_4_O_9_ samples prepared by SPS can provide increased *σ* values [33,46,48,54,59,60,61], while higher substitutions lead to decreased *σ* values [47,54,59]. In general, the lower *σ* values measured for all Fe and Ni added samples can be attributed to the more resistive secondary phases and the increase in porosity, caused by the Ca_3_Co_4_O_9_ phase decomposition. Additionally, for the Ni-added samples, the lower *σ* values could also be explained by the partial substitutions with more Ni^2+^ (than Ni^3+^) ions, which would produce crystal distortions due to the ionic radii mismatches from the conductive CoO_2_ planes of the Ca_3_Co_4_O_9_ phase, creating more scattering centers, and resulting in lower *σ* values, as it was also found in other similar works from literature [33,47]. Following the basic equation for the electrical conductivity, σ=neμ [62] (where *n*, *e*, and *μ* are the charge carrier concentration, in this case, holes, the electron charge, and the holes’ mobility, respectively) and using the polycrystalline-doped Ca_3_Co_4_O_9_ system holes concentration in the range 2–4·10^20^ cm^−3^ [63,64] and the holes mobility of ~1 cm^2^V^−1^s^−1^ [63], one immediately gets *σ* values in the range ~30–60 Scm^−1^, supporting the values from the current measurements (~30–40 Scm^−1^) for the pure matrix compositions and for the 3 and 6Fe_1ST samples (best 1ST *σ* values), in agreement with similar results from the literature [33].

The thermopower values for all 1ST sintered samples are positive (p-type holes conduction) and increase almost linearly with temperature (Figure 4II). The *α* values are significantly affected by the Fe and Ni additions, likely due to the partial incorporation into the Ca_3_Co_4_O_9_ phase, as reported elsewhere, for various substitution cases [47,48,59,60,61,63,65]. In general, if we assume that the progressive decrease in *σ* values, for both Fe and Ni additions (from 3 to 9 vol.%) is caused by progressively decreasing *n* values, one can easily explain the opposite trends seen for the *α* values, namely, a progressive increase from 3 to 9 vol.% additions. Such a correlation is typical for thermoelectric materials [47,54,59]. The highest α values are measured for the 3 and 6 Ni_1ST samples (188 and 177 μVK^−1^ at 975K). These samples simultaneously possess the highest relative densities obtained in this work.

The PF values calculated from the measured *σ* and *α* values are plotted in Figure 4III. All samples show a similar behavior, the PF values increasing proportionally with temperature. Besides the pure matrix compositions (105 μWm^−1^K^−2^) and the selected Co additions, the samples showing the best PF values are 3 and 6Fe_1ST (80 μWm^−1^K^−2^) and 3Ni_1ST (90 μWm^−1^K^−2^), at the highest measured temperature. The highest PF value of the pure matrix composition (105 μWm^−1^K^−2^) is higher than other reported value from the literature, measured for similar samples (70 and 80 μWm^−1^K^−2^) [42,46]. The latter witnesses that high-quality materials were used in the present work to verify the proposed approaches.

**Figure 5:**
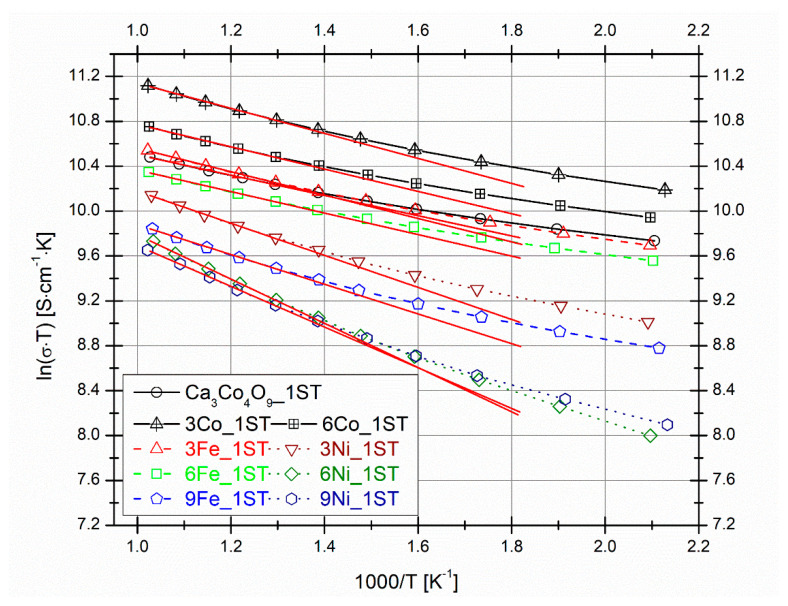
*ln(σ·T)* vs. *1000/T* (Arrhenius) plots for all 1ST sintered samples. The red lines represent linear fittings at high temperatures for activation energy (*E_a_*) calculations.

The sequence of *ln(σT)* vs. *1000/T* (Arrhenius) plots for all 1ST sintered samples is shown in Figure 5. The activation energies *E_a_* in each case (see Table 1) were calculated from the linear fittings (the slopes of the red lines; knowing that *σT∝exp(-Ea/k_B_T)*, where *k_B_* is the Boltzmann constant) performed at high temperatures (five measurement points from ~775–975 K), indicating also the typical variable range hopping electrical conduction behavior from all samples at these temperatures [66]. The *E_a_* values calculated for the selected 1ST sintered Ca_3_Co_4_O_9_ samples with 3 and 6 vol.% Co additions (used only for comparison; not shown in Table 1) are 96 meV and 86 meV, respectively. These *E_a_* values for the best Co added samples (reported previously) are higher than the 78 meV value calculated for the pure matrix composition, probably because of the additional scattering sites created by the presence of the secondary Co_3_O_4_ phase from these samples. On the other hand, similar *E_a_* values to those of the reference matrix samples from this work can also be found elsewhere in the literature, for similar samples and cases [59,63,66]. The addition of both Fe and Ni generally results in an increase of the activation energy, provided by the formation of less conductive phases with the more pronounced thermally activated character of the electronic transport.

The 2ST sintered samples present quite different compositional, morphological, microstructural, and electrical properties, and are discussed in the next section.

### 3.2. The Samples Processed under the 2ST Route

#### 3.2.1. Compositional and Morpho-Structural Evolution

As for the 1ST case, the abbreviations, phase compositions, and relative densities for all 2ST sintered samples are presented in Table 2. As a preview, relatively high densities are expected to translate in improved TE properties, especially in larger electrical conductivity values. On the other hand, the equally large number of secondary phases, as compared to 1ST case, is also observed, and expected to have a decisive impact on the TE properties.

**Figure 6:**
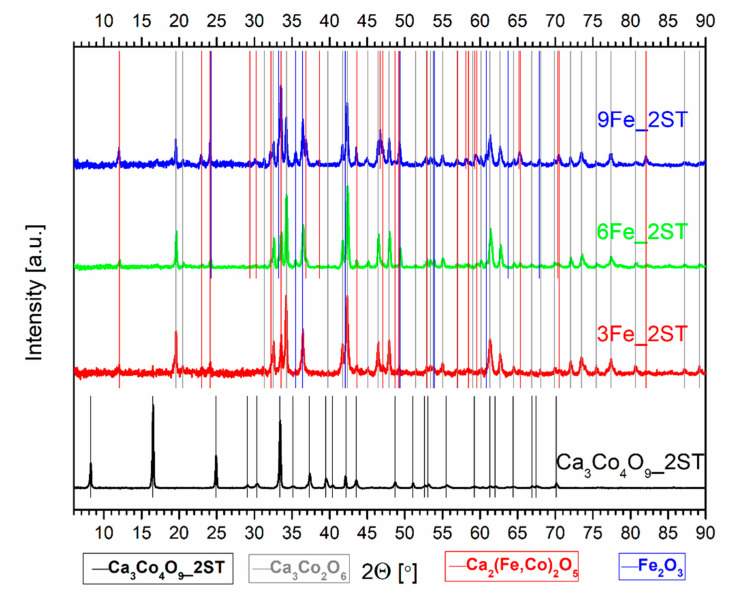
Normalized XRD patterns of the 2ST sintered reference matrix and Fe added samples. The vertical lines of different colors mark the main identified phases.

**Figure 7:**
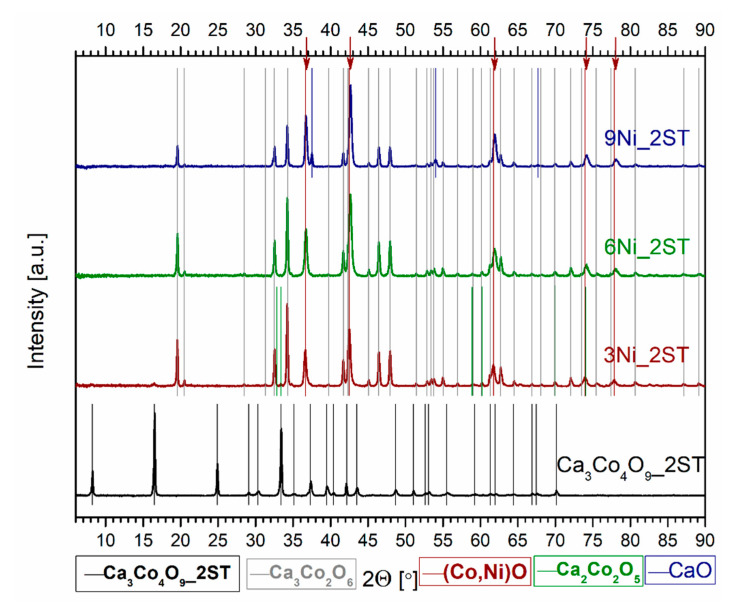
Normalized XRD patterns of the 2ST sintered reference matrix and Ni added samples. The vertical lines of different colors mark the main identified phases.

Figure 6 and Figure 7 show the XRD patterns for the 2ST sintered reference matrix and for all Fe and Ni added samples, respectively. The longer processing time (>80 h, in total), including the high-temperature step, results in fairly different phase compositions, compared to those found in the 1ST sintering case. These phases are marked in the corresponding figures by the various vertical lines of different colors, following the legends from the base of the figures. The arrows from the upper part from Figure 7 mark the visible peak displacements (to higher 2θ values), identified for the CoO phase, in agreement with the variable Ni containing (Co,Ni)O solid solutions, found in all Ni added samples. First of all, the sought-out Ca_3_Co_4_O_9_ phase (pure and/or doped) was only detected in the reference matrix composition, without any impurity phases, marked in both figures by the vertical black lines, in agreement with the work of Masset et al. [29] and with other literature references [44,45]. The slightly narrower peaks observed for this phase suggest some degree of texturing, probably due to more pronounced grain growth, expected to occur during the 2ST sintering route.

The thermoelectric Ca_3_Co_4_O_9_ phase could not be detected in neither the 2ST sintered Fe and Ni added samples, where the main phases have been found to be Ca_3_Co_2_O_6_ and brownmillerite Ca_2_(Co,Fe)_2_O_5_ [67], for the Fe additions, and Ca_3_Co_2_O_6_ and (Co,Ni)O, for the samples with Ni additions. Some minor reflections corresponding to the Ca_3_Co_4_O_9_ phase are slightly visible in Figure 7, only for the 3Ni_2ST samples. Additionally, the long processing time from this second route also facilitates the segregation of iron oxides, found in all Fe added samples in relatively similar, small amounts (Table 2). Similar trends are also reported elsewhere in the literature [54,59], for SPS textured samples with high Fe substitutions. On the other hand, the Ni additions promote the segregation of CoO (and, additionally, CaO, for the 9Ni_2ST samples), followed by the formation of (Co,Ni)O solid solutions, similar to the 1ST case. This is again provided by the total solubility between CoO and NiO at 1173 K [55], the resulting solid solutions having higher stability than Ni doped Ca_3_Co_2_O_6_.

**Figure 8:**
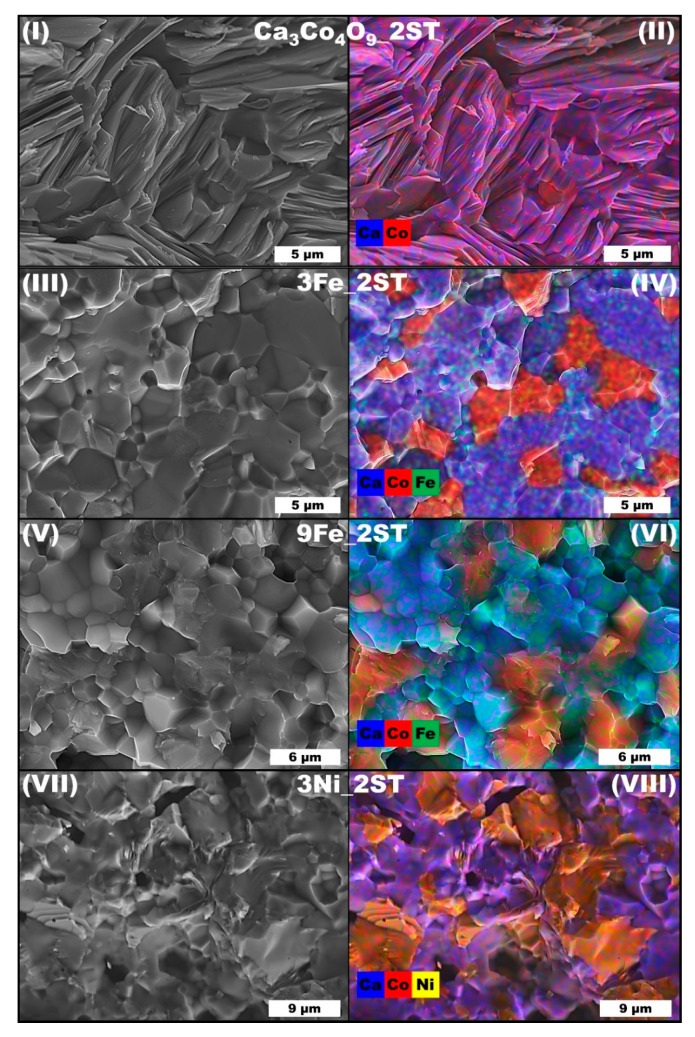
Representative SEM micrographs (**I**,**III**,**V**,**VII**) and EDS maps (**II**,**IV**,**VI**,**VIII**) of selected 2ST sintered samples (fractures).

As expected, the morphological and microstructural evolution of all Fe and Ni added samples (Figure 8) is complex and fairly distinct from the 1ST case. Quite similar results have been published previously for Co additions [49]. The reference matrix composition is the only 2ST sintered sample showing a typical Ca_3_Co_4_O_9_ microstructure and grain morphology. The reference sample also presents the typical plate-like crystallites, as seen for the previous 1ST route, except here the grains grew 4 or 5 times larger (~5 μm) and the porosity decreased significantly (~20%). When Fe and Ni particles are added to the Ca_3_Co_4_O_9_ matrix and the 2ST sintering cycle is applied, the resulting microstructures change drastically to much denser ones, and the grain morphologies become completely different than in the equivalent 1ST case, as shown in Figure 8III,V,VII. The oxidation of metallic Fe and Ni particles is integral, as expected, and the number of resulted phases found in all samples is higher than for the 1ST sintering case. The pore-filling effect of the added metallic particles is hard to be distinguished from the large grain growth, promoted by the 2ST sintering cycle.

The Fe added samples apparently present the densest microstructures from all those obtained in this work. The grains found in the representative Fe added samples are also more irregular in shape and size (Figure 8III). As for their composition, the representative EDS map from Figure 8IV (6Fe_2ST samples) unambiguously shows that the smaller grains are richer in Co (Fe doped Ca_3_Co_2_O_6_), while the larger ones are richer in Ca (Fe-containing brownmillerite). The 9Fe_2ST samples (Figure 8VI) show the same trends, with even smaller grains and larger Fe-rich areas, probably belonging to the Fe_2_O_3_ phase. The Ni added samples present similar microstructural and morphological features (Figure 8VII and VIII) to those seen in the Fe added samples (low porosity, irregular grains). In contrast, for these samples, Ni is mostly present in the CoO phase, similar to the 1ST case. In fact, (Co,Ni)O is actually the major phase found in the 6 and 9Ni_2ST samples (Table 2 and Figure 7).

The intimate interplay between all the characteristic features discussed up to this point is expected to have a complex effect on the electrical performances of the 2ST sintered Fe and Ni added samples.

#### 3.2.2. Electrical Performance

**Figure 9:**
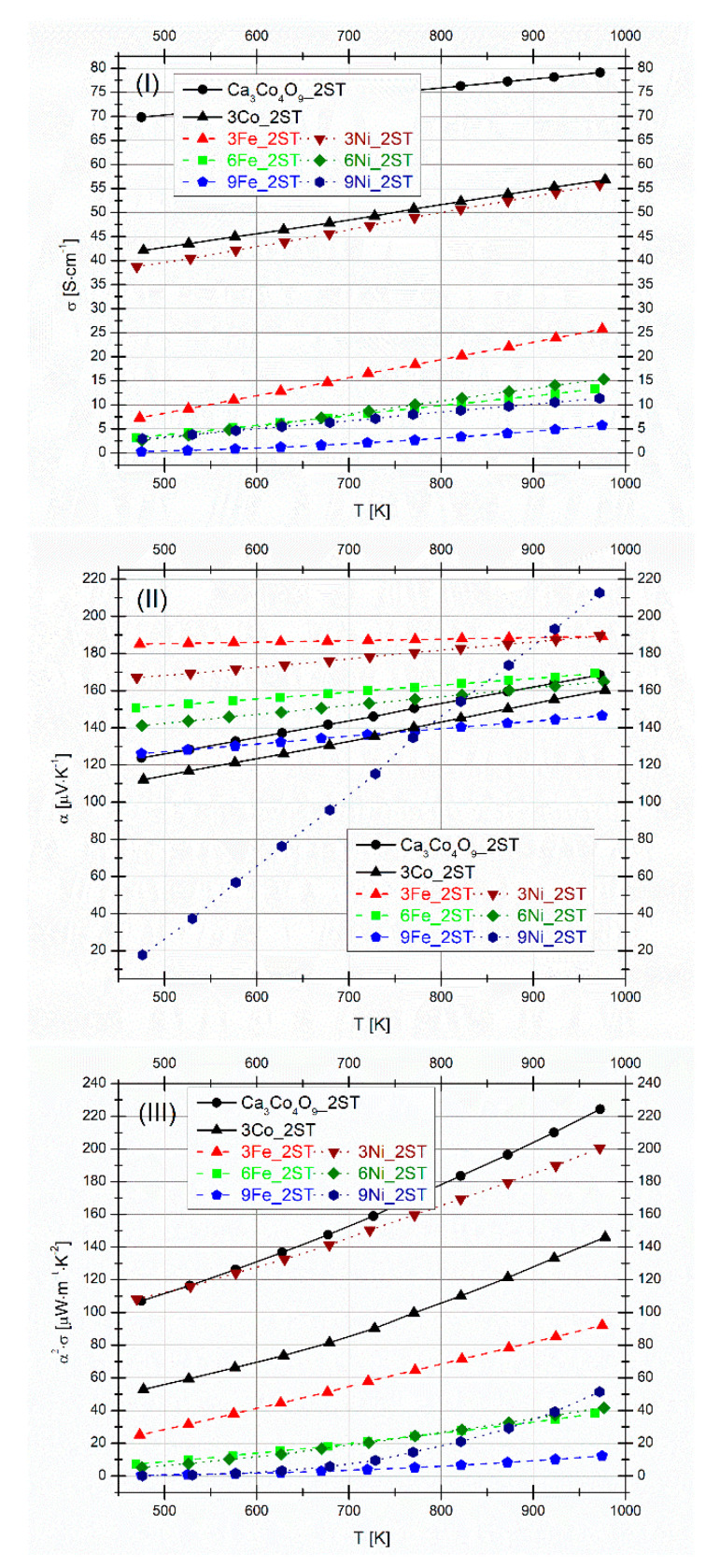
Electrical conductivity (**I**), Seebeck coefficient (**II**) and power factor (**III**) for 2ST sintered samples. The results for selected Co additions are presented for comparison.

The evolution of *σ*, *α,* and *α^2^·σ* with temperature can be seen in Figure 9I–III, respectively. The previously obtained results [49] for selected Co additions (3 vol.%) are displayed for comparisons. All conductivity values increase almost linearly with temperature, showing a typical semiconducting behavior in the whole measured temperature range. The reference Ca_3_Co_4_O_9_ sample exhibits the largest σ values (~70–80 Scm^−1^) owing to “ideal” phase composition and larger density achieved. These values are around the best values reported in literature, for high-density/SPS textured Ca_3_Co_4_O_9_ samples [42,46,54,57,58]. Since the Fe and Ni additions result in immediate phase decomposition even for relatively low addition contents, the electrical conductivities of Fe and Ni added samples are notably below that of the reference matrix composition. Still, the 3Ni_2ST sample demonstrates relatively high σ values (~56 Scm^−1^ at 975 K), which cannot be explained solely based on the observed phase composition (Table 2). In fact, the electrical conductivity of polycrystalline Ca_3_Co_2_O_6_ at high temperatures is at least 6–8 times lower [68] than that measured for the 3Ni_2ST sample, which contains ~76 wt.% of this phase (Table 2). This difference is even larger for lower temperatures. At the same time, the crystal structure of Ca_3_Co_2_O_6_ is highly anisotropic, and electrical conductivity values measured for single crystals along the c-axis direction reach values of up to 79 Scm^−1^ at 975 K [69]. Specific conditions of the Ca_3_Co_2_O_6_ phase formation in the studied composite formulations, namely, a continuous phase transformation of the Ca_3_Co_4_O_9_ phase matrix, promoted by the presence of nickel particles, oxidizing to nickel oxide, may result in a somewhat intermediate scenario between compacted polycrystalline samples and single crystal. However, it is not sufficient to explain the much less temperature-activated character of the 3Ni_2ST sample’s conductivity, as compared to Ca_3_Co_2_O_6_ [68,69]. Some guidelines can be obtained from the Figure 8VIII, showing a clear percolation of Co-rich phase, in opposition to isolated Co-rich grains in the case of 3Fe_2ST sample (Figure 8IV), possessing notably lower electrical conductivity. While any positive contribution of the (Co,Ni)O phase to the relatively high electrical conductivity of the 3Ni_2ST sample is rather unlikely [70], also taking into account that this phase is present in higher amounts in the 6Ni_2ST and 9Ni_2ST samples possessing lower conductivity, one might attribute the relatively high σ values observed for 3Ni_2ST to the presence of Ca_2_Co_2_O_5_. The layered Ca_2_Co_2_O_5_ phase shows electrical conductivity values in the range 50–60 Scm^−1^ at 400–950 K [71], comparable to those measured for 3Ni_2ST. In general, despite the large density values calculated for all samples, the lower *σ* values (<26 Scm^−1^) measured for most Fe and Ni added samples can be attributed to the high number of resistive secondary phases, found by XRD and confirmed by SEM-EDS analyses. However, the results observed for the 3Ni_2ST sample suggest that the implemented approach can still be considered promising, if lower Ni additions are used.

The values of the Seebeck coefficient for all samples are positive (holes conduction) and increase linearly with temperature, in the whole measured temperature range, following different slopes (Figure 9II). The *α* values also vary considerably for all samples and, although it may appear counterintuitive, the samples with the highest *σ* values also present the highest *α* values (the 3Ni and 3Fe_2ST samples). Significant variations of the Seebeck coefficient values in transition metal-substituted Ca_3_Co_4_O_9_ samples were also observed in other works from literature [47,48,59,60,61,63,65]. The results clearly indicate that the behavior of the Seebeck coefficient with composition is mostly determined by the complex phase composition. Similarly to the case of Co additions [49], the large *α* values found for the 3Ni and 3Fe_2ST samples can be explained by the large amounts of Ca_3_Co_2_O_6_ phase in these samples, which is known for its large high-temperature *α* values [68].

The resulting PF values can be seen in Figure 9III. Besides the reference sample measuring the largest PF values (225 μWm^−1^K^−2^), the next samples showing high PF values are 3Ni, 3Co, and 3Fe_2ST (~200, ~150, and ~90 μWm^−1^K^−2^, respectively), which are better than some of the best-reported values found in literature, for equivalent samples [46]. Still, this comparison is not entirely correct, since these samples no longer represent the Ca_3_Co_4_O_9_-based composites. The large PF value measured for the reference sample is equal or slightly higher than other best-performing high-density/textured samples of the same composition, found in literature [33,42,59].

**Figure 10:**
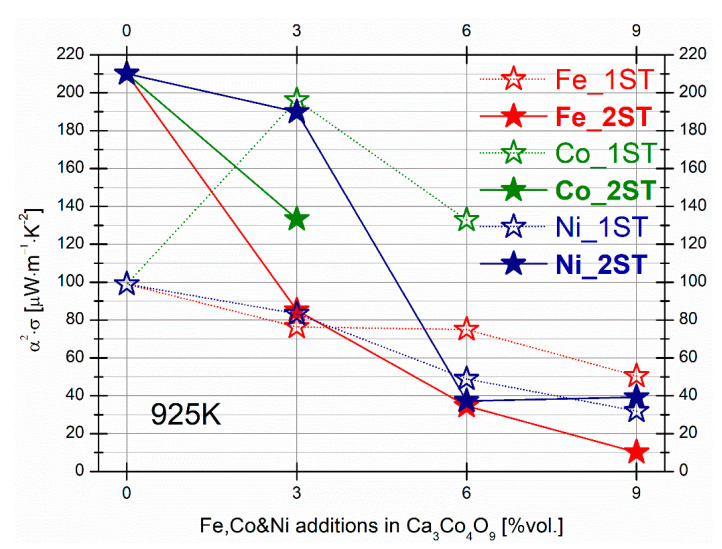
Compositional dependence of the power factor at 925 K, for the 1ST and 2ST sintered samples. The results for selected Co additions are shown for comparison.

The compositional dependence trends of the PF values from 925 K for both 1ST and 2ST sintered samples can be seen in Figure 10. From this plot, it is clear that the highest PF values (between around 190 and 210 μWm^−1^K^−2^) from this work are achieved for Ca_3_Co_4_O_9__2ST and 3Ni_2ST, mostly due to the high density and less severe impact on the phase composition in the case of minor Ni addition. Higher Fe and Ni additions promote the formation of more resistive secondary phases, which lead to lower PF values in the corresponding samples.

From Figure 10 it becomes obvious that addition levels of 3% and lower are essential for more drastic improvements in thermoelectric performances, provided by the different particularities and strongly correlated nature of this semiconducting ceramic material.

This work intended to analyze the relevant effects provided by the separate Fe and Ni metallic particles additions on the electrical counterpart of the TE performance of Ca_3_Co_4_O_9_-based materials, inspired by our previous successful proof-of-concept, for the case of cobalt additions. Although the results appear rather pessimistic in terms of the observed performance, they clearly show the existence of different pathways to tailor the phase composition of the Ca_3_Co_4_O_9_-based materials, while also highlighting the necessity in more controllable approaches. Partially encouraging results achieved for the 3Ni_2ST samples suggest that the amount of transition metal additions should be lowered. The kinetics of the pore-filling effects can also be adjusted by the size of the Fe and Ni particles and by selection of more appropriate processing conditions.

## 4. Conclusions

This work continues the previous study on the electrical performance improvement strategy of Ca_3_Co_4_O_9_-based materials, by transition metal additions and employment of different sintering schemes, this time involving a combined approach using separate metallic Fe and Ni particles additions in the Ca_3_Co_4_O_9_ structure and sintering in one and two stages, in air. Novel composite materials with the general formula (1 − x)Ca_3_Co_4_O_9_/xFe (and xNi) (x = 0%, 3%, 6% and 9% vol.) have been prepared through a modified Pechini solution-based synthesis route and sintered in two different ways, producing low density (the one-step cycle, 1ST) and high density (the two-step cycle, 2ST) ceramic composites. Selected samples from each composition have been characterized by measurements of electrical conductivity (σ), Seebeck coefficient (α), and power factor (PF), between 475 and 975 K, and related to their respective composition, morphology, and microstructure. The 1ST sintered samples presented high porosity (31–62% of *ρ_th_*) and the highest PF values of 80 and 90 μWm^−1^K^−2^ have been recorded for the 3% vol. Ni and 3 and 6% vol. Fe-added composites, respectively, very close to some of the best reported values in literature. In contrast, the 2ST case produced high-density samples and the best PF values have been measured for the 3% vol. Ni added composites (200 μWm^−1^K^−2^). Generally, both 1ST and 2ST sintered composites presented rather complex phase composition and microstructures, which led to only minor electrical performance improvements, compared to the pure matrix samples, but very close to or even better than some of the best reported values in literature, for similar cases and materials.

## Figures and Tables

**Table 1:** Abbreviations, phase composition, relative densities, and activation energies for all one-stage (1ST) sintered samples.

Prepared Materials	Abbreviation	RIR-Estimated Phase Composition, wt.%	Experimental Density *ρ_exp_*, g/cm^3^	*ρ_exp_/ρ_th_* *, %	*E_a_* **, meV
Ca_3_Co_4_O_9_	Ca_3_Co_4_O_9__1ST	Ca_3_Co_4_O_9_ (100)	2.62	56	78
Ca_3_Co_4_O_9_ + 3% vol. Fe	3Fe_1ST	Ca_3_Co_4_O_9_ (95); (Fe,Co)_3_O_4_ (5)	2.38	–	90
Ca_3_Co_4_O_9_ + 6% vol. Fe	6Fe_1ST	Ca_3_Co_4_O_9_ (83); (Fe,Co)_3_O_4_ (9); (Fe,Co)_2_O_3_ (8)	1.68	–	83
Ca_3_Co_4_O_9_ + 9% vol. Fe	9Fe_1ST	Ca_3_Co_4_O_9_ (83); (Fe,Co)_3_O_4_ (7); (Fe,Co)_2_O_3_ (10)	1.50	–	114
Ca_3_Co_4_O_9_ + 3% vol. Ni	3Ni_1ST	Ca_3_Co_4_O_9_ (67); Ca_3_Co_2_O_6_ (28); CoO (5);	2.85	60	123
Ca_3_Co_4_O_9_ + 6% vol. Ni	6Ni_1ST	Ca_3_Co_4_O_9_ (39); Ca_3_Co_2_O_6_ (43); CoO (18)	3.09	62	170
Ca_3_Co_4_O_9_ + 9% vol. Ni	9Ni_1ST	Ca_3_Co_4_O_9_ (35); Ca_3_Co_2_O_6_ (42); CoO (23)	2.90	57	156

* *ρ_th_* of the composites are calculated using the theoretical density values of the RIR-estimated phases and their respective amounts (a simple mixing rule); ** *E_a_* is the activation energy of the electronic transport, calculated from the electrical conductivity data (see below).

**Table 2:** Abbreviations, phase composition, and relative densities for all two-stage (2ST) sintered samples.

Prepared Materials	Abbreviation	RIR-Estimated Phase Composition, wt.%	Experimental Density *ρ_exp_*, g/cm^3^
Ca_3_Co_4_O_9_	Ca_3_Co_4_O_9__2ST	Ca_3_Co_4_O_9_ (100)	3.74
Ca_3_Co_4_O_9_ + 3% vol. Fe	3Fe_2ST	Ca_3_Co_2_O_6_ (74); Ca_2_(Fe,Co)_2_O_5_ (22); Fe_2_O_3_ (4)	4.16
Ca_3_Co_4_O_9_ + 6% vol. Fe	6Fe_2ST	Ca_3_Co_2_O_6_ (62); Ca_2_(Fe,Co)_2_O_5_ (29); Fe_2_O_3_ (9)	3.88
Ca_3_Co_4_O_9_ + 9% vol. Fe	9Fe_2ST	Ca_3_Co_2_O_6_ (32); Ca_2_(Fe,Co)_2_O_5_ (60); Fe_2_O_3_ (8)	3.55
Ca_3_Co_4_O_9_ + 3% vol. Ni	3Ni_2ST	Ca_3_Co_2_O_6_ (73); Ca_2_Co_2_O_5_ (9); (Co,Ni)O (18)	4.12
Ca_3_Co_4_O_9_ + 6% vol. Ni	6Ni_2ST	Ca_3_Co_2_O_6_ (48); (Co,Ni) O (52)	4.63
Ca_3_Co_4_O_9_ + 9% vol. Ni	9Ni_2ST	Ca_3_Co_2_O_6_ (42); (Co,Ni)O (52); CaO (6)	4.07

## Data Availability

Data sharing not available.
